# New Insights Into the Regulation of γδ T Cells by BTN3A and Other BTN/BTNL in Tumor Immunity

**DOI:** 10.3389/fimmu.2018.01601

**Published:** 2018-07-11

**Authors:** Juan-Luis Blazquez, Audrey Benyamine, Christine Pasero, Daniel Olive

**Affiliations:** ^1^INSERM, U1068, Centre de Recherche en Cancérologie de Marseille (CRCM), Immunity & Cancer, Institut Paoli-Calmettes; Aix-Marseille Université UM105, CNRS UMR 7258, Marseille, France; ^2^Aix-Marseille Université (AMU), Médecine Interne Hôpital Nord, Assistance Publique Hôpitaux de Marseille (AP-HM), Marseille, France; ^3^Imcheck Therapeutics, Marseille, France; ^4^Immunomonitoring platform, Institut Paoli-Calmettes, Marseille, France

**Keywords:** γδ T cells, butyrophilins, BTN3A, tumor immunity, immunotherapy

## Abstract

Recent findings in the immunology field have pointed out the emergent role of butyrophilins/butyrophilin-like molecules (BTN/BTNL in human, Btn/Btnl in mouse) in the modulation of γδ T cells. As long as the field develops exponentially, new relationships between certain γδ T cell subsets, on one hand, and their BTN/BTNL counterparts mainly present on epithelial and tumor cells, on the other, are described in the scientific literature. Btnl1/Btnl6 in mice and BTNL3/BTNL8 in humans regulate the homing and maturation of Vγ7+ and Vγ4+ T cells to the gut epithelium. Similarly, Skint-1 has shown to shape the dendritic epidermal T cells repertoire and their activation levels in mice. We and others have identified BTN3A proteins are the key mediators of phosphoantigen sensing by human Vγ9Vδ2 T cells. Here, we first synthesize the modulation of specific γδ T cell subsets by related BTN/BTNL molecules, in human and mice. Then, we focus on the role of BTN3A in the activation of Vγ9Vδ2 T cells, and we highlight the recent advances in the understanding of the expression, regulation, and function of BTN3A in tumor immunity. Hence, recent studies demonstrated that several signals induced by cancer cells or their microenvironment can regulate the expression of BTN3A. Moreover, antibodies targeting BTN3A have shown *in vitro* and *in vivo* efficacy in human tumors such as acute myeloid leukemia or pancreatic cancer. We thus finally discuss how these findings could help develop novel γδ T cell-based immunotherapeutical approaches.

## γδ T Cell Subsets and Related BTN/BTNL Proteins

### γδ T Cell Subsets

Arising from the same common multipotent double negative precursor than the αβ T cells, and differentiated earlier in the thymus, γδ T cells comprise a heterogeneous group of cells that are considered to be a link between innate and adaptive immunity.

The main characteristic that defines the γδ T cells is the expression of its distinctive TCR composed by a γ-chain and a δ-chain, that is called γδ TCR. In humans, 0.5–16% of all CD3+ cells in adult peripheral blood and organized lymphoid tissues (thymus, spleen, tonsil, and lymph nodes) are γδ T cells, they usually represent less than 5% in tongue and reproductive tract and 10–30% in intestine (1, 2). In adult mice, 1–4% of the entire T cell compartment in thymus, secondary lymphoid organs and lung are γδ T cells. Higher numbers of γδ T cells are found in other mucosal sites, reaching until 10–20% of all T cells in female reproductive organs (3), 20–40% of the intestinal intraepithelial T cells (4), and 50–70% of skin dermal T cells (2, 5, 6).

Like B cells and αβ T cells, γδ T cells have an RAG-mediated rearranged antigen receptor by the combination of V (variable), D (diversity), and J (joining) gene segments. In human, there are only a few Vγ (Vγ 2,3,4,5,8,9) and Vδ (Vδ 1,2,3,4,6,7,8) germline genes that can be used to rearrange (7). Nonetheless, the CDR3 loop of Vδ-chain shows a high degree of diversity thanks to multiple D gene segments. In addition, the insertion or loss of N-nucleotides during the junctional diversification process further enriches this diversity (8). Available Vγ and Vδ genes are not randomly used. Thus, certain Vδ chains tend to pair almost exclusively with another Vγ chain (like Vγ9 and Vδ2 in humans), and some γδ T cells expressing certain Vγ and Vδ genes preferentially reside in well-defined tissue locations (Table 1).

**Table 1:** γδT cell subsets and tissue locations in human and mouse.

Species	V gene segment pairing	V(D)J diversity	Tissue location	References
Human	Vδ1-T cells	High	Thymus, spleen, dermis, liver (with Vδ3), and gut epithelia (with Vδ3)	(9, 10)
	Vγ9Vδ2-T cells	Intermediate	Main peripheral blood γδ T population (paired with Vγ9)	(9, 11)
	Vδ3-T cells	High	Liver, higher numbers in chronic viral infections and leukemia, gut epithelia	(9, 10, 12)
	Vδ4, Vδ6, Vδ7, Vδ8-T cells		Peripheral blood of lymphoma patients	(13)

Mouse	Vγ1/Vγ4 γδ-T cells	High	Predominant in spleen	(14)
	Vγ7-T cells	Intermediate	Gut epithelia (paired with Vδ4, Vδ5, and Vδ6)	(14)
	Vγ4/Vγ6-T cells	Intermediate	Lungs	(14)
	Vγ6Vδ1-T cells	Invariant	Main population in reproductive organs	(14)
	Vγ5Vδ1-T cells (dendritic epidermal T cells)	Invariant	Major subset in mice skin	(15)
	Diverse	High	Adult thymus	(10)
	Diverse	High	Lymph node	(10)
	Vγ1Vδ6/3, Vγ4 and Vγ6-T cells	Intermediate	Liver	(10)

In contrast to conventional αβ T cells, the antigen recognition mechanism in γδ T cells is not MHC-restricted. Indeed, γδ T cells seem to be implicated in the recognition of different antigens than αβ T cells. While αβ T cells recognize non-self-peptide fragments restricted by MHC molecules, γδ T cells can recognize unconventional antigens as stress molecules (MICA and MICB), non-peptidic metabolites of isoprenoid biosynthesis, heat-shock proteins, and so on (12).

As cytotoxic CD8+ T cells do, γδ T cells can elicit a broad cytotoxic activity against infected and transformed cells. This cytotoxic activity is based on death receptor/ligand (Fas/FasL) signals and perforin/granzyme to destabilize cellular integrity (12). γδ T cells also secrete various cytokines (16) and chemokines including proinflammatory Th1-like cytokines such as IFN-γ and TNF-α in order to activate several immune mediators (dendritic cells and Th1), arrest proliferation, and kill target cells (17). Thanks to these cytotoxic properties, γδ T cells highlight among other immune mediators, along with CD8+ T and NK cells, as a powerful tool for cancer immunotherapy.

### The BTN/BTNL Protein Families

The butyrophilins (BTN) and butyrophilin-like (BTNL) genes are part of the immunoglobulin superfamily. They are structurally related to the B7 proteins, which comprise co-stimulatory (B7-1, ICOS, etc.) and co-inhibitory (PD-L1, PD-L2, B7-H3, etc.) molecules involved on T lymphocytes regulation. The seven human BTN genes are clustered in the MHC class I region of chromosome 6 (18, 19) into three phylogenetically related subfamilies: BTN1, BTN2, and BTN3, in humans. The BTN1 subfamily consists only in the BTN1A1 gene, whereas the BTN2 and BTN3 subfamilies have three genes: BTN2A1, BTN2A2, and BTN2A3 pseudogene, and BTN3A1, BTN3A2, and BTN3A3. The BTN proteins show high structural homology, with BTN1, BTN2, and BTN3 subfamilies sharing the 50% of their amino acid identity. The homology between the BTN2 and BTN3 is even closer, reaching on average 80% identity (20). The high homology rates observed suggest that the BTN genes have undergone tandem duplication. Although further, the BTNL family still shares considerable homology (on average 40% identity) to the BTN-family members. Five identified BTNL genes are encoded in the human genome. BTNL2, the best-known member of this family, is clustered with the BTN genes in the MHC class I region of chromosome 6, while the chromosome 5 harbors the coding sequences of BTNL3, BTNL8, and BTNL9. The genomic sequence of SKINTL pseudogene is found in the chromosome 1.

**Figure 1:**
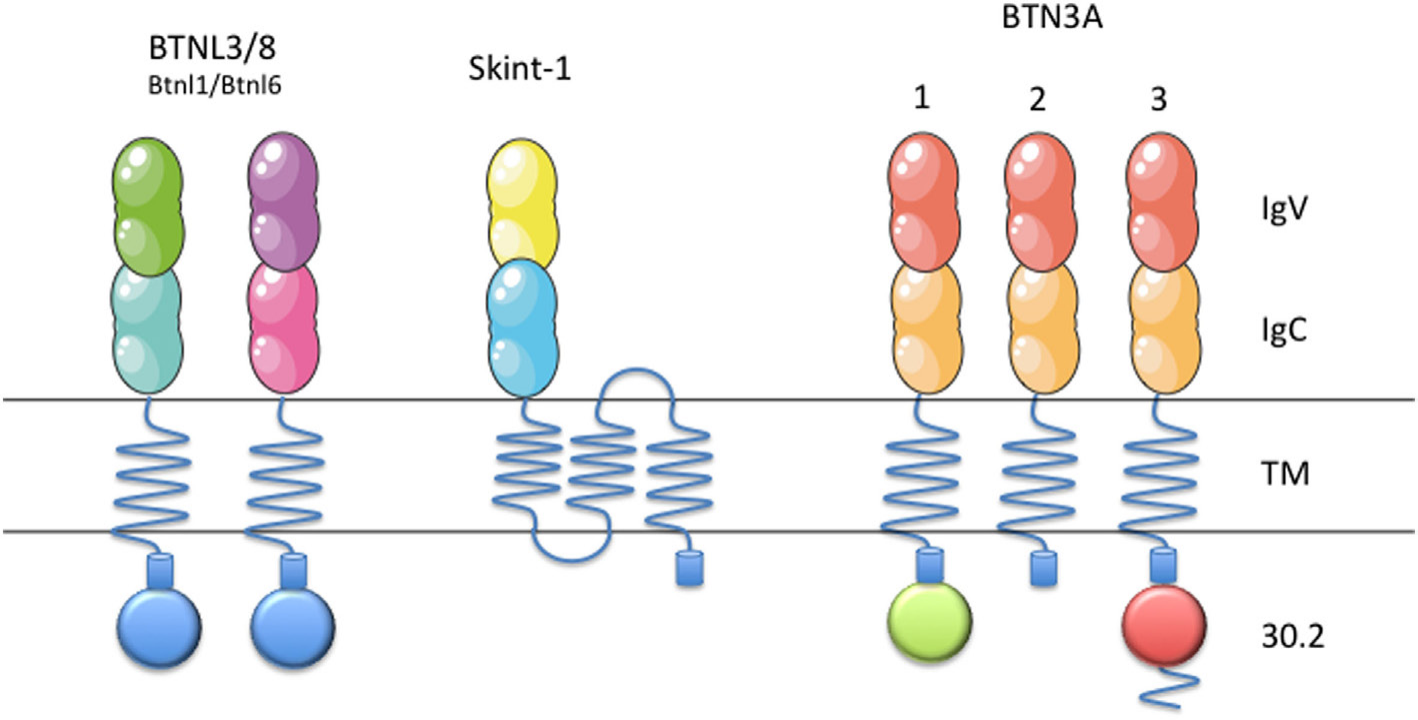
Schematic representation of BTN3, Btnl1, Btnl6, BTNL3, BTNL8, and Skint-1. Schematic representation of BTN/BTNL molecules in mice (Btnl1, Btnl6, and Skint-1) and humans (BTN3, BTNL3, and BTNL8), that have been shown to regulate specific γδ T cell subsets. Each subfamily member contains an extracellular, N-terminal IgV, and a membrane-proximal IgC domain connected to a single-pass transmembrane domain. BTN3A1 and BTN3A3 contain intracellular B30.2 domains, as well as BTNL3 and BTNL8, which is missing in BTN3A2. This figure was created in part using graphics from Servier Medical Art (https://smart.servier.com/) with permission.

In the mouse genome, however, only two Btn proteins are found: Btn1a1, who plays a role in the regulation of milk fat globule secretion (21), and Btn2a2. Both genes are clustered on chromosome 13, and are orthologs of human BTN1A1 and BTN2A2, respectively. Eight different murine Btnl genes have been described so far (Btnl1, 2, 4, 5, 6, 7, 9 and skint-1). Two of them are predicted to be pseudogenes (Btnl5 and Btnl7). Six of them are located in the same genomic region, at the MHC class II locus (chromosome 17), while Btnl9 is the only Btnl gene found on chromosome 11, whereas the Skint-1 (Figure 1) gene is encountered on chromosome 4. Given their high extracellular identity, Btnl9 and Btnl2 are thought to be orthologs of human BTNL molecules. Recent advances in the field have pointed out the role of BTN3, Skint-1, Bntl1/Btnl6, and BTNL3/BTNL8 as key immune regulators of γδ T cells in humans and mice (Figure 1) (22–26).

## Regulation of Specific γδ T Cell Subsets by BTN/BTNL Proteins

### Skint-1 and Dendritic Epidermal T Cells (DETCs)

**Figure 2:**
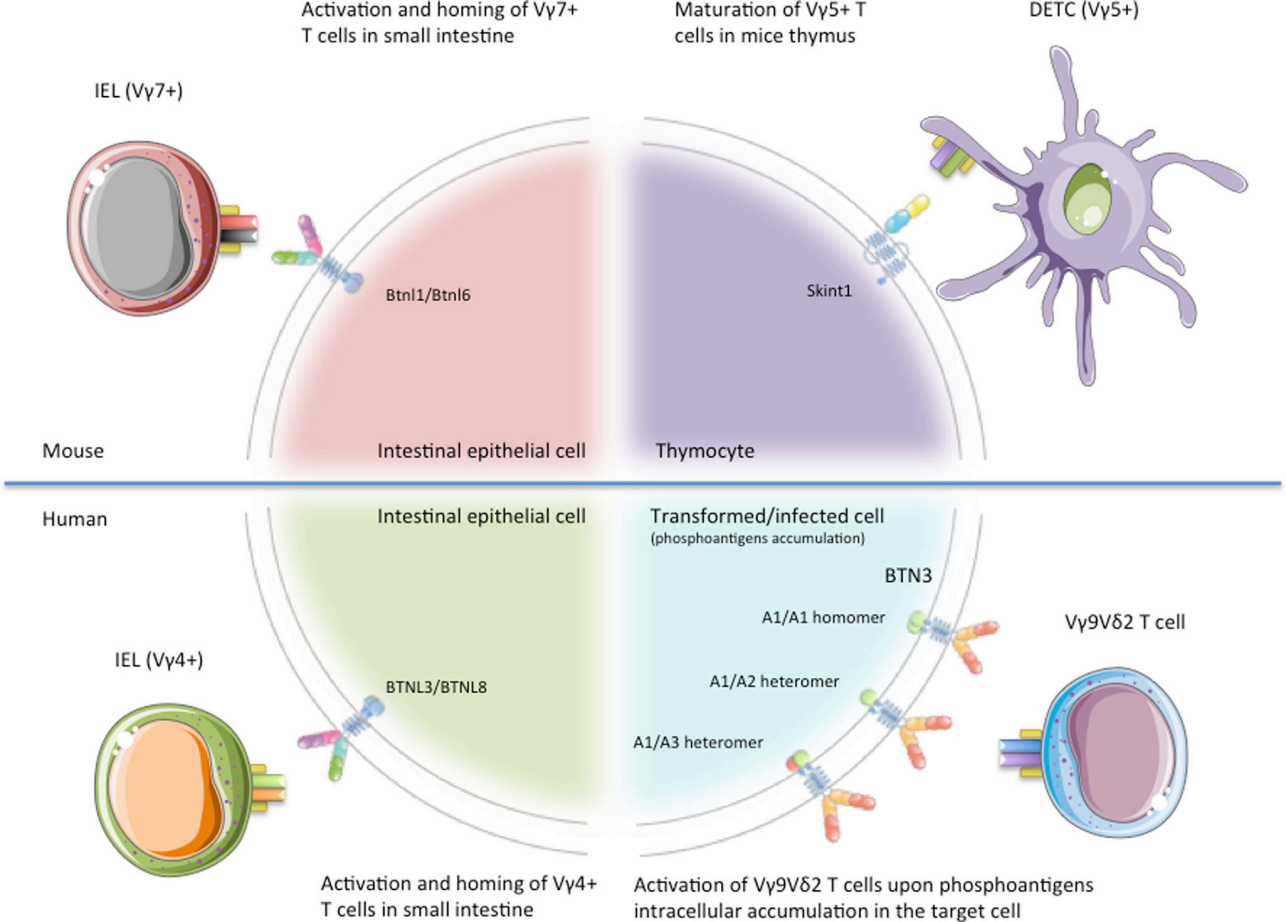
Schematic representation of the different γδ T cell subsets regulated by Btn/Btnl proteins. Representation of the published functions of BTN/BTNL proteins on specific γδ T cell subsets. Skint-1 has been shown as critical for the maturation of Vγ5Vδ1+ dendritic epidermal T cells (DETCs) in mice thymus. Btnl1/Btnl6 promote Vγ7+ intraepithelial lymphocytes (IELs) maturation and expansion within mice small intestine, whereas BTNL3/BTNL8 regulate the activation of Vγ4+ IEL in human gut epithelium. Finally, BTN3A molecules play a mandatory role in the recognition of tumor or stressed cells by human Vγ9Vδ2 T cells. This figure was created in part using graphics from Servier Medical Art (https://smart.servier.com/) with permission.

The study of the T cell repertoire in mice with a Skint-1−/− genetic background pointed out the relevance of the interaction between Btn/Btnl proteins and γδ T cells, as these mice do not develop canonical Vγ5Vδ1+ DETCs (27, 28). Skint-1 is a transmembrane protein without any known ortholog in humans (Figure 1), but it shows a high degree of homology with a subfamily of BTNL molecules, which are conserved in humans (25, 29, 30). Skint-1 is expressed at detectable levels exclusively by keratinocytes and thymic epithelial cells, where it promotes the IFN-γ production and TCR hyporesponsiveness of DETC progenitors (Figure 2) (31, 32). Mature DETCs exist in a semiactivated state under homeostatic conditions. This activation is explained, at least in part, by a constitutive TCR engagement through ligand recognition on surrounding keratinocytes within this tissue *in vivo* (33). Nevertheless, while a DETC TCR ligand was expressed on the surface of keratinocytes at the wound edge in FVB-Tac mice (a substrain of FVB mice, harboring a mutation in Skint-1, specifically deficient for Vγ5Vδ1 DETCs), Skint-1 was not able to directly bind the DETC TCR, neither detected on the surface of keratinocytes (19, 25, 31). Thus, although Skint-1 expression is fundamental for the development of canonical Vγ5Vδ1+ DETCs, the underlying mechanisms by which Skint-1 promotes the maturation of these cells remain poorly understood. However, DETC tetramers did inhibit wound closure *in vivo* (34) suggesting that Skint-1 might not be the ligand, or at least the only ligand, of the DETC TCR in keratinocytes. It has to be noted that direct binding between γδ TCRs and any Btn/Btnl has not been described so far in the scientific literature.

Intriguingly, Skint-1 was not readily expressed at the cell surface of HEK293 cells transfected with WT Skint-1 (28). This finding suggests that an accessory protein might help Skint-1 to properly localize at the cell membrane compartment. Unfortunately, this accessory protein has not been identified so far.

### Btnl1/Btnl6 and Vγ7+ IELs in Mice, BTNL3/BTNL8 and Vγ7+ IELs in Humans

In mice, several Btnl proteins are only expressed at protein level in the intestinal epithelium, concretely on enterocytes of the small-intestinal villus epithelial cells (25, 26). In this way, the expression of Btnl1 by small-intestinal villi at an early time point in life was recently found to critically and selectively promote Vγ7+ intraepithelial lymphocytes (IELs) maturation and expansion within the tissue (Figure 2) (26). The first evidence came from the study of IEL populations on four different strains of Btnl1−/− mice, where Vγ7+ IEL numbers were depleted by ~90%, with Vγ7+ Vδ4+ cells almost ablated. The specificity of the interaction between Vγ7+ IELs and Btnl1 was emphasized by the fact that Btnl4−/− mice displayed no overt defects in any major IEL subset.

In 2016, Lebrero-Fernandez et al. reported an enhancement on cell surface expression of Btnl1 on Btnl1-transfected MODE-K cells when these cells were concomitantly transfected with Btnl4 and Btnl6 (35). In the same way, Btnl1 greatly enhanced the expression of Btnl6 on the cell surface *in vitro* (26). Conversely, co-transfecting Btnl1 or Btnl6 did not augment the cell surface expression of Btnl4. These results match with the fact that Btnl4−/− mice displayed no overt defects in any major IEL subset.

Vγ7+ IELs co-cultured with MODE-K stably expressing Btnl1+ Btnl6 cells overexpress the T cell activation marker CD25, downregulate the TCR and CD122 expression levels and show higher levels of granulocyte–macrophage colony-stimulating factor, CCL4, and IFN-γ *in vitro* (26). Likewise, it was observed that human gut epithelial cells as well express BTNL3 and BTNL8, and that concomitant expression of BTNL3 + BTNL8 induces selective TCR-dependent responses of human colonic Vγ4+ cells (Figure 2) (26). When HEK293 cells were transfected with BTNL3, BTNL8, or BTNL3 + BTNL8, only Vδ2− cells co-cultured with HEK293 cells co-expressing BTNL3+ BTNL8 undergo a marked TCR downregulation (26). Among all the γδ T cell subsets included on the Vδ2− population, only those expressing Vγ4 effectively downregulated TCRs in co-cultures with L3 + L8 cells. Similar to Skint-1, investigations have failed to report direct γδ IEL-TCR–BTNL molecule interactions up to date. As for BTNL1 and BTNL6 described above, neither BTNL3 nor BTNL8 protein was efficiently expressed on cells transfected with their respective genes, unless both were co-expressed (36).

### Vγ9Vδ2 T Cells and BTN3A Proteins

Due to the pairing restrictions observed in the formation of the γδ TCR, the Vδ2 chain can be combined almost exclusively with Vγ9 forming the Vγ9Vδ2-TCR. Vγ9Vδ2 T cells represent the major γδ T cell subset in the human peripheral blood, with values ranging from 50 to 95% of γδ T cells. These immune mediators, which have been only found in humans and non-human primates and are evolutionarily conserved in selected species like alpaca (*Vicugna pacos*) (37), stand out as having the capacity to “sense” several infected and malignant cells. This Vγ9Vδ2 T cell reactivity has been associated with intracellular accumulation of organic pyrophosphate-containing molecules, also called phosphoantigens (pAgs) (38). These molecules can be produced by microbes, such as hydroxy-methyl-butyl-pyrophosphate (HDMAPP, also known as HMBPP), a microbial intermediate of the 2-C-methyl-d-erythritol 4-phosphate (MEP) pathway or can be synthetized endogenously, such as isopentenyl pyrophosphate (IPP), an intermediate of the mevalonate pathway in mammal cells that can accumulate in transformed cells during tumorigenesis (39).

After these findings, nitrogen-containing bisphosphonates (N-BPs) like Zoledronic acid have emerged as drugs that, indeed, can raise up the intracellular levels of IPP by the inhibition of farnesyl diphosphate synthase (FPPS) (40, 41), thus facilitating the activation of human Vγ9Vδ2 T cells against tumor cells. In the same way, several synthetic analogs of HMBPP-like bromohydrin pyrophosphate (BrHPP) have been synthetized up to date, showing all of them potent stimulation of Vγ9Vδ2 T cells (42).

Although the Vγ9Vδ2-TCR of Vγ9Vδ2 T cells is sufficient for pAg recognition (38), the cell–cell contact is mandatory for the proper Vγ9Vδ2 T cell activation against cancer cells [although some pAgs, like BrHPP can activate Vγ9Vδ2 T cells without necessity of cell–cell contact (43, 44)], suggesting the presence of a “pAg presenter molecule” in target cells. In addition, none non-primate cell can stimulate the Vγ9Vδ2 T cells (45), indicating that this antigen-presenting molecule (APM) is characteristic of primates (like Vγ9Vδ2 T cells). On the other side, Vγ9Vδ2 T cell activation relies on the expression of the Vγ9Vδ2-TCR as Jurkat cells became reactive against pAgs intracellular accumulation in their targets when they were transfected with Vγ9Vδ2-TCR (46). Finally, Harly and coworkers demonstrated the role of the butyrophilin-3A (BTN3A, also called CD277) subfamily of proteins as key mediators of pAg signaling (47).

The second part of this review will be focused on the peculiar antigenic activation process of human Vγ9Vδ2 T cells through BTN3A proteins, and consequently on the mechanism of tumor cell recognition by Vγ9Vδ2 T cells. Recent publications have shed new light on the mode of action of BTN3A.

## Focus on BTN3A Role on the Activation Process of Human Vγ9Vδ2 T Cells

### BTN3A1/A2/A3 Isoforms

Due to the recent discovery of these proteins, most concepts about these molecules remain still obscure. BTN3A subfamily of molecules belongs to the B7 co-stimulatory family of molecules. BTN3A subfamily is composed by three members in humans: BTN3A1, BTN3A2, and BTN3A3 (Figure 1) (18). They exhibit 95% identity between them in their extracellular part, thus forming a monophylogenetic group along with the BT-related members (48). Each subfamily member contains an extracellular, N-terminal IgV, and a membrane-proximal IgC domain connected to a single-pass transmembrane domain. BTN3A1 and BTN3A3 both contain an intracellular B30.2 domain, which is not found in BTN3A2. After its B30.2 domain, BTN3A3 has a unique cytoplasmic tail of 70 amino acids.

All the three isoforms, BTN3A1, BTN3A2, and BTN3A3, after treatment with the 20.1 agonist monoclonal antibody (mAb) can stimulate Vγ9Vδ2 T cells, thus activating them through mechanisms involving decreased mobility (47) and a multimerization of BTN3A molecules (49). These findings suggest the involvement of their extracellular domains in the activation process. Nonetheless, only the BTN3A1 isoform, and to a much lesser extent BTN3A3, can trigger the activation of Vγ9Vδ2 T cells upon phosphoantigen burst (47).

The way BTN3A1 presents pAgs to Vγ9Vδ2 T cells has remained controversial over the last years. The receptor for BTN3A, similar to the receptor for other BTNs or B7-H4, remains to be identified. The very first model of pAgs presentation hypothesized that BTN3A might present pAgs directly to Vγ9Vδ2 T cells *via* its extracellular IgV domain (50). This model fails to explain why BTN3A2 is unable to activate Vγ9Vδ2 T cells, taking into account that the IgV domains of BTN3A1 and BTN3A2 share the 95% of their amino acid sequence. However, the binding of pAgs to the BTN3A IgV domain was not confirmed by other groups. Harly and coworkers pointed out the importance of the B30.2 intracellular domain of BTN3A1 in the activation process of Vγ9Vδ2 T cells by swapping the B30.2 intracellular domains of BTN3A1 and BTN3A3 (47). Thus, the chimeric BTN3A3 protein carrying the BTN3A1 B30.2 intracellular domain became stimulatory. In fact, the activation levels reached by Vγ9Vδ2 T cells when co-cultured with cells expressing the BTN3A3 chimeric protein were higher than those obtained when Vγ9Vδ2 T cells were cultured with cells expressing BTN3A1 WT. Conversely, cells transfected with chimeric BTN3A1 protein carrying the BTN3A3 B30.2 were no longer able to activate Vγ9Vδ2 T cells. Based on these results, a second model for pAgs presentation to Vγ9Vδ2 T cells was proposed. In this model, supported by several articles, BTN3A1 might act as an indirect antigen presentation molecule. Thus, Vγ9Vδ2 T cells would not recognize directly the pAg binding to the IgV domain of BTN3A, but they might recognize the conformational changes triggered in BTN3A1 by the binding of pAgs to its B30.2 domain. Even more recent data focusing on the BTN3A1 B30.2 domain show that only the B30.2 intracellular domain of BTN3A1 can directly bind pAg through a positively charged surface pocket under physiological conditions. Several basic residues along this binding pocket including histidines (His351 and His378), arginines (Arg412, Arg418, and Arg469), and a lysine (Lys393) were probed essential to allow pAgs binding as they provide a highly positive charged environment complementary to the negative charge of the pyrophosphate moiety of pAgs. By contrast, in the B30.2 domain of BTN3A3, a single amino acid change in the position 351 from histidine (in BTN3A1) to arginine (in BTN3A3) prevents the binding of pAgs to the surface pocket and, thus, impedes the subsequent conformational changes that, ultimately, will achieve the effective activation of Vγ9Vδ2 T cells (51).

As we have seen above, the BTN3A2 isoform lacks the B30.2 intracellular domain, reason why this receptor might be unable to activate Vγ9Vδ2-T cells upon pAgs intracellular accumulation (Figure 1). Surprisingly, several reports highlighted the importance of BTN3A2 and BTN3A3 expression in the activation of Vγ9Vδ2 T cells against their targets. Indeed, BTN3A1, A2, and A3 KD/KO cell lines were unable to activate Vγ9Vδ2-T cells as much as their WT counterparts upon pAgs intracellular accumulation (36, 52, 53). Recently, Vantourout et al. showed that HEK cells KO for BTN3A1 alone, failed to activate Vγ9Vδ2 T cells, even in the presence of BTN3A2 and/or BTN3A3, confirming that BTN3A1 is strictly required for Vγ9Vδ2 T cell activation. However, HEK cells expressing BTN3A1 but neither BTN3A2 nor BTN3A3 did not activate Vγ9Vδ2 T cells. This fact suggests that BTN3A2 and BTN3A3 are somehow involved in the activation mechanism of Vγ9Vδ2 T cells. In 2014, Riano et al. pointed out through transductant experiments that gene(s) on Chr6 in addition to BTN3A1 are mandatory for PAg-mediated activation of Vγ9Vδ2 T cells (54).

### BTN3A Homo- and Heteromers

Last published evidences shed some light to this controversy. Adams et al. showed that BTN3A1 tends to form heterodimers with BTN3A2, as well as BTN3A1 homodimers in a lesser extent, in a native cellular environment. During their experiments, the stability of BTN3A1/A2 heterodimers was higher than those of BTN3A1 homodimers. This observation suggests that BTN3A1 tends to pair with BTN3A2 in a physiological context (55). Further confirmation of BTN3A1–A2 interaction arrived in 2018. In their work, Vantourout and coworkers showed that BTN3A2 is able to complex with BTN3A1 in an IgC-dependent manner. They also achieved to co-immunoprecipitate BTN3A3 along with BTN3A1 (Figure 2). Moreover, they demonstrated that BTN3A1 was intrinsically inefficient at cell surface localization when transfected alone in a BTN3 triple KO background. Strikingly, surface expression of BTN3A1 was greatly enhanced by co-transfecting BTN3A2 or BTN3A3 with BTN3A1. Thus, BTN3A1 transfected alone largely colocalized with the endoplasmic reticulum (ER). Nevertheless, BTN3A1 showed reduced ER colocalization in cells cotransfected with BTN3A2. These findings demonstrate that BTN3A2 helps BTN3A1 to properly localize at the cell membrane, where it exerts its action (36). It should also be noted that in their functional assays, double BTN3A2/A3 KO cells were unable to activate Vγ9Vδ2 T cells, suggesting than BTN3A1 alone is unable to mediate Vγ9Vδ2 T cell activation without A2 and/or A3. Reconstitution with either BTN3A2 or BTN3A3 in combination with BTN3A1 in BTN3 KO cells recovered the ability of cells to activate Vγ9Vδ2 T cells, which means that BTN3A2 and BTN3A3 might have a redundant role in the activation process of Vγ9Vδ2 T cells.

Once again, similarly to the behavior of Btnl1 and Btnl6 in mice, as well as BTNL3 and BTNL8 described above, BTN3A1 was not efficiently expressed unless co-expressed with BTN3A2 or BTN3A3. As BTN3A2 and BTN3A1 did, Btnl1/Btnl6 coexpression reduced their colocalization with the ER. BTNL3 and BTNL8 behave similarly in order to be co-expressed at the cell membrane compartment. Strikingly, this cellular mechanism, which allows the localization of one protein to the cell membrane only when its partner protein is also expressed, seems to be conserved among several members on the BTN/BTNL families and between rodents and humans (26), even though these proteins are not predicted to be orthologs.

### Other BTN3A1 Interactors

From 2015, Periplakin was also identified as an important interactor of BTN3A1 in the activation process of Vγ9Vδ2 T cells (52). Rhodes and coworkers demonstrated that the cytoskeletal adaptor protein Periplakin interacted with a di-leucine motif, C-terminus to the B30.2 domain of BTN3A1. However, Periplakin did not interact with BTN3A2 or BTN3A3, which do not contain the di-leucine motif. Interestingly, Periplakin depleted cells did not activate Vγ9Vδ2 T cells upon N-BPs treatment, suggesting a key role for Periplakin binding in the activation process of Vγ9Vδ2 T cells.

Last insights into the complex relationships governing the interactions of the actin cytoskeleton with BTN3A proteins came out in 2016, when Sebestyen et al. highlighted the critical role of RhoB small GTPase in the stabilization of BTN3A1 in the membrane, as well as its attachment to the actin cytoskeleton (56). Thus, they show that RhoB localizes mainly to the nucleus in cells resistant to Vγ9Vδ2 T cells-mediated lysis and, by contrast, it preferentially localizes to the cell membrane in cells that are sensitive to Vγ9Vδ2-T cells lysis. Besides that, it is also shown that the inhibition of RhoB GTPase activity on target cells with C3 transferase dramatically reduced the activation levels of Vγ9Vδ2TCR+ T cells by LCL48 cells. In addition, RhoB was selectively excluded from nuclear areas in cells that are able to activate Vγ9Vδ2 TCR+ T cells, like leukemic blast of acute myeloid leukemia. Conversely, healthy stem cells from the same donor, which express RhoB mainly in the nucleus, did not activate Vγ9Vδ2 TCR+ T cells. Thus, exclusion of RhoB from the nucleus might be a cellular event which confers sensitivity to lysis by Vγ9Vδ2 TCR+ T cells. Taken together, these results suggest a critical role of Periplakin and RhoB in the activation mechanism of Vγ9Vδ2 T cells mediated by BTN3A.

### BTN3A Functions in Other Immune Cells

Activating γδ T cells may not be the only function of BTN3A molecules. Some studies have highlighted the role of BTN3A in the regulation of TCR-mediated αβ T cells responses. In fact we demonstrated in 2004 using the BTN3A mAb 20.1 that the molecule was largely expressed on most immune cells (48). A first study showed that the engagement of BTN3A1 expressed in co-stimulatory artificial APC, coated with CD3 mAb, through its counter-receptor expressed on CD4 T cells resulted in a decrease of CD3/CD28-dependent proliferation of CD4 T cells (57). The effect of BTN3 engagement itself on αβ T cells has been confirmed by two studies. Anti-BTN3A 232.5 mAb cross linking on CD4 and CD8 T cells induced BTN3A3 phosphorylation and inhibited CD3- and IL-2-induced T cell activation (58). Artificial APC consisting of CD3/CD28/BTN3A 20.1 mAb coated beads enhanced co-stimulation-induced CD4 T cell proliferation and cytokine production through enhanced TCR signaling (59).

In contrast with CD4 T cells, BTN3A2 is the most abundant transcript found in NK cells compared to BTN3A1 and BTN3A3 isoform, and the only one able to inhibit NK cells-induced cytokine production. As BTN3A2 is devoid of B30.2 domain, one could postulate that BTN3A2 could be a decoy receptor. This hypothesis was further supported by the decrease of NKp30-induced cytokine production following the specific engagement of BTN3A2 but not BTN3A1 triggering on KHYG-1 transfected cells (59).

When engaged on monocytes and immature dendritic cells (iDCs) with plate-coated anti-BTN3A 19.5 or 20.1 mAbs, BTN3A protected cells from apoptosis and induced the expression of co-stimulatory and APMs. BTN3A stimulation with mAb synergized with toll-like receptor stimulation to increase chemokine and proinflammatory cytokine production (60). These data suggested that BTN3A subfamily could enhance and amplify inflammatory signals that are initiated by other receptors. On THP-1 cells [human monocytic cell line derived from acute myeloid leukemia (AML) patient], depletion of BTN3A1 inhibited the cytoplasmic nucleic acid- or virus-triggered activation of IFN-β production, suggesting that BTN3A1 may be a novel regulator of type I IFN responses (61).

To note, cross linking of BTN3A mAbs is needed to obtain a biological effect on monocytes, iDCs, and αβT cells and this effect is the consequence of direct engagement of BTN3A expressed by responder immune cells. In marked contrast, soluble BTN3A mAbs can, in the absence of any other stimuli, sensitize the tumor cell to mediate Vγ9Vδ2 T cell activation.

## Current Advances on BTN3A Expression and Regulation in Tumors

### BTN3A Expression in Tumors

BTN3A members are widely expressed in various tumors of hematological origin such as acute myeloid leukemia (48, 62) and solid tumors such as breast, colon (52, 63), ovarian (64), and more recently in gastric cancer (65) and pancreatic ductal adenocarcinoma (PDAC) (53) (Table 2). Immunohistochemical analysis of PDAC tissue microarray confirmed BTN3A expression in all the tested tumor samples, whereas BTN3A expression was either absent or barely detectable in control pancreatic tissue. Regarding the isoforms, BTN3A2 was the most expressed isoform in primary AML blasts (62). In addition, BTN3A overexpression and a dominant expression of the BTN3A2 isoform were strongly associated with a poor prognosis, in gastric cancer and PDAC (62, 65). This overexpression of BTN3A2 gene was associated with an increased proliferation and invasion of gastric cancer cells. Given that BTN3A2 lacks the B30.2 intracellular domain and could potentially be considered as a decoy receptor, its predominant expression in AML blasts or other tumors could constitute an immune escape mechanism to Vδ9Vδ2 T cell recognition. Another study showed that epithelial BTN3A expression evaluated by immunohistochemistry was significantly associated with better prognosis in high grade serous epithelial ovarian cancer patients, and correlated with higher density of infiltrating T cells (64). The role of BTN3A isoforms may be complex and BTN3A function in tumors could be regulated by the combinations of isoforms.

**Table 2:** BTN3A expression in tumors.

Cancer	Method	Observations/correlation with prognosis	Reference
Cancer cell lines	FC	Cell surface BTN3A expression on: T, B or monocytic leukemia cell lines Solid tumor (breast, pancreas, and ovary) cell lines	(48)

Ovarian cancer	IHC	BTN3A2 observed on epithelial cells, some tissue cores, and stroma Correlated with CD3+ immune infiltrate High BTN3A2 associated with increased OS and disease free progression	(64)

Breast cancer	IHC	Increased BTN3A1/BTN3A3 staining in tumors sections compared to normal epithelium	(52)

AML blasts	FC qRT-PCR WB	BTN3A cell surface expression BTN3A2 most abundant isoform both at transcriptional and protein level	(62)

Colorectal cancer	qRT-PCR WB IHC	Three isoforms detected by WB BTN3A1 detected on epithelial cells and TAFs	(63)

Gastric cancer	Exome array analysis	BTN3A2 associated with poor prognosis, increased proliferation and invasion of gastric cancer cell lines	(65)

PDAC	IHC FC qRT-PCR WB	BTN3A epithelial expression in PDAC, associated with invasiveness BTN3A2 most abundant isoform both at transcriptional and protein level High BTN3A2 transcript associated with reduced OS	(53)

*AML, acute myeloid leukemia; FC, flow cytometry; IHC, immunohistochemistry; OS, overall survival; PDAC, pancreatic ductal adenocarcinoma; qRT-PCR, real-time quantitative reverse transcription PCR; TAF, tumor-associated fibroblast; WB, western blot*.

### BTN3A Regulation by the Tumor Microenvironment

BTN3A is upregulated under T_H_1 stimulation on the surface of normal tissue namely human vein endothelial cells (48). Other studies have demonstrated that certain factors of the tumor microenvironment are able to regulate BTN3A expression (Table 3): inflammatory cytokines and hypoxia-associated mediators such as VEGF, CCL3, and IL-10 upregulate BTN3A expression in ovarian cancer (57). In a recent study, we have shown that hypoxic and metabolic stress increase BTN3A2 isoform transcript in pancreatic cell lines and patient-derived xenograft-cell lines. In addition, soluble BTN3A isoforms including soluble BTN3A1 are found in the supernatants of pancreatic cell lines and in the plasma of PDAC patients (53). The soluble isoform results from BTN3A shedding that is in part MMP-dependent, similar to that previously described for the NKG2D ligands MICA/B (66). Soluble BTN3A and sBTN3A1 were associated with a decreased overall survival in PDAC patients, and consequently could represent new attractive prognosis biomarkers. The mechanism underlying the pro-tumoral effect of sBTN3A has to be clarified. One hypothesis could be that sBTN3A may prevent Vδ9Vδ2 T cells from exerting their cytotoxic activity on tumor cells. This feature of soluble form is also shared with other B7 family members such as CTLA-4 and PD-L1 and has been linked to patient prognosis (67, 68). The soluble forms of proteins are usually generated by proteolytic cleavage of the membrane-bound form, as in the case of soluble tumor necrosis factor receptor (69) and sB7-H3 (70), or by translation of alternative spliced mRNA, as in the case of sB7-2 (71, 72), as well as sCTLA-4 (67, 68) and sB7-H3 (73). Increasing evidences suggest that sPD-L1 is a prognostic biomarker associated with aggressive disease in malignant tumor, such as multiple myeloma (74), diffuse large B-cell lymphoma (68), renal cell carcinoma (75), and ovarian cancer (76). In the same way, sBTN3A may provide new biomarkers easily detectable in clinical practice.

**Table 3:** Factors driving BTN3A regulation in the tumor microenvironment.

	Observations	Reference
Inflammatory cytokines: TNFα, IFNγ, CCL3, and IL-6	BTN3A upregulation on DCs and HUVECs	(48, 57)
Hypoxia-associated mediators such as VEGF, PlGF, and IL-10	BTN3A upregulation in ovarian cancer	(53, 57)
	BTN3A2 transcript upregulation in PDAC	
Nutrient deprivation	BTN3A2 transcript upregulation in PDAC	(53)
Soluble BTN3A isoforms	Plasma sBTN3A associated with decreased OS	(53)

*DCs, dendritic cells; HUVECs, human vein endothelial cells; OS, overall survival; PDAC, pancreatic ductal adenocarcinoma*.

### Interactions Between γδ T Cells and BTN/BTNL Proteins: Therapeutic Opportunities

γδ T cells, both Vδ1+ and Vδ2+ subsets, have been shown to infiltrate a wide range of solid tumors, as well as blood cancers such as follicular lymphoma, AML Burkitt’s lymphoma. Strikingly, intratumoral γδ T cells have emerged as the most favorable prognostic immune population among many cancer types (77). Their capacities to secrete effector cytokines and to kill tumor cells make them attractive as new immunotherapeutic targets. Lawand et al. have recently reviewed the main features of γδ T cell subsets in cancer (78). However, multiple signals within the tumor microenvironment can influence the functional outcome (79). The major challenge remains thus to determine how to specifically boost the anti-tumor effects of γδ T cells. Two synthetic drugs, the pAg BrHPP and N-BP Zoledronate activate γδ T cells *in vitro* and in clinical trials *in vivo*. However, their utility in clinics has been dampened by their bad pharmacokinetics and pharmacodynamics properties which favor their local accumulation at the bone surface and rapid renal clearance (80). New approaches have to be considered to optimize the use of γδ T cells in therapeutics. As reviewed here, BTN and BTNL have demonstrated a potent immunomodulatory role for certain γδ T cell subsets. The T-cell-stimulatory and inhibitory activity of some BTN/BTNL proteins suggests that they might be taken into account as novel targets for checkpoint inhibition (like CTLA-4, PD1, PD-L1, etc.) to potentially target γδ T cells (81).

### BTN3A and Vγ9Vδ2 T Cells

BTN3A has been shown as crucial for mediating Vγ9Vδ2 cytolytic functions against tumor cells. We have developed anti-BTN3A antibodies which selectively promote an active (mAb 20.1) or inactive (mAb 103.2, 108.5) BTN3A conformation on the cell surface, and might broadly open the Vγ9Vδ2-T cells immunotherapy field for cancer and autoimmune diseases. Notably, the 20.1 mAb was shown to sensitize primary AML blasts and to circumvent their resistance to allogeneic Vγ9Vδ2 T cells lysis. This effect induced the clearance of primary leukemic blasts from the bone marrow *in vivo* in an NSG human AML-xenografted mouse model (62). The 20.1 mAb was also shown to boost BTN3A-mediated Vγ9Vδ2 T cells cytolytic functions against PDAC even under hypoxic conditions, overcoming this stress-related characteristic of the PDAC microenvironment (53). Harly et al. have also shown that 20.1 mAb induced the activation of Vγ9Vδ2 T cells against a wide range of tumor cell lines (47). A recent study highlighted that 20.1 mAb differentially activates Vγ9Vδ2-TCR clonotypes, and that the responsiveness strongly depends on CDR3 sequences of the TCR (82).

Vγ9Vδ2 T cells were also shown to be able to kill colorectal cancer cell lines exposed to Zoledronate, this effect being partly related to BTN3A1 expression and its cellular re-distribution, in the membrane and cytoskeleton-associated fraction (63). Cross linking of BTN3A on glioblastoma-derived cell lines increased IFN-γ secretion by Vγ9Vδ2 T cells (83).

As discussed above, BTN3A2 was the most expressed paralog along the BTN3A proteins in several AML (62), PDAC cell lines and primary tumors (53), and gastric cancer (65). Interestingly, the 20.1 antibody stands out for its capacity to stimulate Vγ9Vδ2 T cells independently on the predominant BTN3A paralog expressed at the cell membrane. Hence, the dominant expression of BTN3A2 isoform in tumors do not preclude the triggering of BTN3A molecules by the agonist 20.1 mAb and the effective activation of Vγ9Vδ2 T cells. This effect of priming and sensitization of tumor cells to killing by Vγ9Vδ2 T cells would be a first therapeutic approach. We have also shown that soluble BTN3A are increased in the plasma of PDAC patients and could potentially interfere with the tumor killing activity of effector cells. Development of antibodies to mop-up soluble BTN3A could thus be another strategy.

Therefore, agonistic anti-BTN3A antibodies might represent a novel therapeutic opportunity to treat cancer. Conversely, humanized inhibitory anti-BTN3A antibodies might be taken into account as immunosuppressive drugs for the treatment of autoimmune diseases genetically associated with BTN3A, like rheumatoid arthritis or schizophrenia (84).

Oppositely, patients with ulcerative colitis (UC) showed downregulation of BTN1A1, BTN2A2, BTN3A2, and BTN3A3 levels compared to healthy controls (85). This result might be explained by the fact that BTN3A proteins trigger opposite cell responses in Vγ9Vδ2 T cells (co-activation) and conventional T lymphocytes (co-inhibition) (86, 87). For example, BTN3A expression levels, upregulated by tumor microenvironment signals in ovarian cancer, have been suggested to contribute to immune evasion by dampening the activity of infiltrating T cells (57). However, BTN3A2 has been shown to be associated with a good prognosis in patients with ovarian cancer, associated with an increased infiltrate of CD4+ T cells (64). Unfortunately, Vγ9Vδ2 T cell infiltrate was not assessed in this study. We can postulate that BTN3A2 would mediate other effects in other type of immune cells. As an example, a co-stimulatory role has been demonstrated for BTN3A in αβ T cells that could explain different functions depending on the cellular type engaged and the tumor context. BTN3A2 promoter and ORF were related also to gastric cancer (65) and type I diabetes (88), respectively. Besides that, Gene-Based Association Analysis revealed BTN3A3 associations with rheumatoid arthritis, lower risk of relapse in ovarian cancer (89), and so on. Aberrant BTN3A3 methylation patterns were also observed on bipolar disorder and schizophrenia (90). Meta-analysis of GWAS Data also related BTN3A2 and BTN3A1 with schizophrenia susceptibility (91).

### Other BTN/BTNL Proteins

The recently described role of BTNL3 and BTNL8 in the homing and maintenance of a semi activated state on Vγ4+ γδ T cells in the human gut might be relevant for the onset of gut autoimmune diseases like UC and inflammatory bowel disease (85). Conversely, it was observed a downregulation of BTNL2, BTNL3, BTNL8, and BTNL9 mRNA in colon tumors, suggesting a co-stimulatory role for BTNL3/BTNL8 (85). Thus, the modulation of the activation signal coming from BTNL3/BTNL8 to Vγ4+ γδ T cells might be a useful approach to treat both diseases in the future.

## Concluding Remarks

Recent studies have highlighted the expression of BTN3A in tumors and its correlation with patient prognosis. Notably, *in vitro* as well as *in vivo* experiments have opened new perspectives in Vγ9Vδ2 T cell-based immunotherapies and shown the potential of agonists BTN3A mAbs toward enhancing Vγ9Vδ2 T cell anti-tumor functions. In addition, sBTN3A may play significant role in tumor pathogenesis, immune responses, and prediction. The modulation of other BTN/BTNL molecules such as BTNL3/BTNL8 seems also of interest in other pathologies such as colon tumors or autoimmune diseases. Targeting BTN/BTNL could thus represent an attractive strategy, alone or in combination with current therapies (i.e., pAgs and monoclonal antibodies targeting immune-checkpoint).

## Author Contributions

JL-B, AB, and CP wrote/revised the manuscript. DO supervised/revised the manuscript. JL-B, AB, and CP contributed equally to this work.

## Conflict of Interest Statement

DO is the cofounder and shareholder of Imcheck Therapeutics. No potential conflicts of interest were disclosed by the remaining authors.
